# Supplementary material for: Wiskott Aldrich syndrome protein regulates non-selective autophagy and mitochondrial homeostasis in human myeloid cells
Source: eLife. 2020 Nov 2;9:e55547. doi: 10.7554/eLife.55547 (PMC7673780; doi:10.7554/eLife.55547)
Supplement: Figure 1—source data 1. — ^Additional variant in WAS gene (c.391G > A, p.Glu1331Lys). ARPC1B, actin-related protein C1B-deficiency; GT, in vivo gene therapy; PBMCs, peripheral blood mononuclear cells; VCN, vector copy number; WAS, Wiskott Aldrich syndrome. [file elife-55547-fig1-data1.docx]

| **Patient** | **Condition** | **Mutation** | **Pre/ post treatment** | **VCN (PBMCs)** |
| --- | --- | --- | --- | --- |
| P1 | WAS | c.97C>T (p.Q33X) | Pre | - |
| P2 | WAS | c.80T>A (p.Leu27His) | Pre | - |
| P3 | WAS | c.80T>A (p.Leu27His) | Pre | - |
| P1 | WAS | c.97C>T (p.Q33X) | 6 months post GT | 0.78 |
| P1 | WAS | c.97C>T (p.Q33X) | 9 months post GT | 1 |
| P4 | WAS | c.913C>T (p.Gln305*) ^^^ | 3 years post GT | 1.61 |
| P5 | ARPC1B | p.Leu247Glyfs*25 | Pre | - |
| P6 | ARPC1B | c.265A>C | Pre | - |
| P7 | ARPC1B | c.265A>C | Pre | - |
| P8 | ARPC1B | c.265A>C | Pre | - |

Figure 1- source data 1: Molecular details of patient monocyte-derived macrophages used in experiments

^ additional variant in *WAS* gene (c.391G>A, p.Glu1331Lys)

ARPC1B, actin-related protein C1B-deficiency; GT, in vivo gene therapy; PBMCs, peripheral blood mononuclear cells; VCN, vector copy number; WAS, Wiskott Aldrich syndrome
