# Supplementary material for: Wiskott Aldrich syndrome protein regulates non-selective autophagy and mitochondrial homeostasis in human myeloid cells
Source: eLife. 2020 Nov 2;9:e55547. doi: 10.7554/eLife.55547 (PMC7673780; doi:10.7554/eLife.55547)
Supplement: Figure 1—source data 2. — ^Same patient. [file elife-55547-fig1-data2.docx]

| **Experiment** | **Mutation** | **WASp expression (% CD14^+^) Pre/ edited/ gene therapy** | **Vector copy number edited/ gene therapy** |
| --- | --- | --- | --- |
| 1 | c.97C>T^^^ | 1.8/ 65/ 54 | 1/ 1.8 |
| 2 | 1483 Del G | 1.7/ 78/ 70 | 1/ 0.8 |
| 3 | c.97C>T^^^ | 1.9/ 81/ 24 | 1/ 1.75 |
| 4 | T302>C | 0.3/ 90/ 70 | 1/ 0.76 |

Figure 1- source data 2: Molecular details of patient stem cell-derived macrophages used for in vitro WAS correction

WASp, Wiskott Aldrich syndrome protein.

^^^ same patient
