# Supplementary material for: Wiskott Aldrich syndrome protein regulates non-selective autophagy and mitochondrial homeostasis in human myeloid cells
Source: eLife. 2020 Nov 2;9:e55547. doi: 10.7554/eLife.55547 (PMC7673780; doi:10.7554/eLife.55547)
Supplement: Figure 3—source data 1. — Meaningful PSSM scores predictive of possible LIR domains are identified in the range 13–17 (highlighted in grey), with balanced accuracy most optimal at 15 or 16. [file elife-55547-fig3-data1.docx]

| **Protein** | **No. WxxL motifs** | **Max WxxL PSSM score** | **No. xLIR motifs** | **Max. xLIR PSSM score** |
| --- | --- | --- | --- | --- |
| **WASp** | 8 | 10 | 0 | 0 |
| **ARP2** | 5 | 5 | 2 | 15 |
| **ARP3** | 8 | 10 | 1 | 17 |
| **ARPC1A** | 4 | 11 | 0 | 0 |
| **ARPC1B** | 4 | 14 | 0 | 0 |
| **ARPC2** | 4 | 12 | 1 | 14 |
| **ARPC3** | 1 | 7 | 0 | 0 |
| **ARPC4** | 1 | 8 | 0 | 0 |
| **ARPC5** | 1 | 3 | 0 | 0 |

Figure 3- data source 1. Summary of LC3-interating regions of human WASp and ARP2/3

Summary of LC3-interating regions of human WASp and all seven subunits of the ARP2/3 complex as identified using the web resource developed by Kalvari et al. Meaningful PSSM scores predictive of possible LIR domains are identified in the range 13-17 (highlighted in grey), with balanced accuracy most optimal at 15 or 16.
